# Immunotherapy in Autoantibody-Associated Psychiatric Syndromes in Adults

**DOI:** 10.3389/fpsyt.2021.611346

**Published:** 2021-01-28

**Authors:** Niels Hansen, Charles Timäus

**Affiliations:** Department of Psychiatry and Psychotherapy, University of Goettingen, Goettingen, Germany

**Keywords:** autoantibodies, autoimmunity, immunotherapy, psychiatry, symptoms

## Abstract

**Background:** Autoantibody-associated psychiatric syndromes are often distinct from, but might also be part of autoimmune encephalitis. Our article focuses on potential immunotherapy in these patients with a probable autoimmune origin of their psychiatric syndrome.

**Methods:** We searched through PubMed for appropriate articles on immunotherapy in autoantibody-associated psychiatric syndromes between 2010 and 2020 for this narrative review.

**Results:** In line with prior recommendations for autoimmune encephalitis and autoimmune psychosis, we suggest that in patients with a probable autoimmune-based psychiatric syndrome should be given early corticosteroids, intravenous immunoglobulins, or plasmapheresis as first line immunotherapy. If these therapeutic options fail, second-line immunotherapy should be applied within 1 month consisting of rituximab or cyclophosphamide. Maintenance therapy is best for those patients responding to steroids including mycofenolate mofetil or azathioprine. So far, there is evidence from a few retrospective cohort studies supporting the usage of first- and second-line, and maintenance immunotherapies for autoantibody-associated psychiatric syndromes. Some immunological agents are discussed that might exert an effect in autoimmune-based psychiatric syndromes, but the latest evidence is low and derived from case reports or series with autoimmune encephalitis patients.

**Conclusions:** Taken together, the immunotherapeutic landscape for patients with autoantibody-associated psychiatric syndromes is delineated. Our suggestions rely on observational studies in autoantibody-associated psychiatric syndromes and a few placebo-controlled, randomized trials for patients with autoimmune encephalitis and psychosis. Thus, adequate powered, prospective as well as placebo-controlled clinical trials in patients with autoantibody-associated psychiatric syndromes are warranted in order to enlighten efficacy and safety aspects of current and novel therapy strategies.

## Introduction

Encephalitis is an infectious inflammation emerging within the brain that often affects the temporal lobe and requires urgent action to prevent further brain damage. However, encephalitis can be characterized as infectious encephalitis, however it can also have an autoimmune-origin, as in autoimmune encephalitis. Autoimmune encephalitis is a disease characterized by seizures, memory disturbances, or psychiatric features in addition to changes in mesiotemporal or extratemporal brain structures in magnetic resonance imaging (MRI), pleocytosis in cerebrospinal fluid (CSF), focal epileptic potentials or slowing in temporal or extratemporal electroencephalography (EEG) (1, 2). We need to distinguish between autoantibody-positive and negative autoimmune encephalitis, and isolated or partly isolated psychiatric symptoms revealing additional hints for an underlying immunopathology. The differentiation between autoimmune encephalitis and autoantibody-based psychiatric syndromes are not clear and might overlapping. If a psychiatric syndrome is detected and autoimmune encephalitis is diagnosed, this disease entity should be called autoimmune encephalitis as the cause is an encephalitis in its nature, although its presentation may be purely psychiatric. However, if a psychiatric syndrome dominates and no specific criteria of possible or probable autoimmune encephalitis are fulfilled, then any psychiatric syndrome's autoimmune basis must be subjected to further investigations, such as a recent diagnostic approach (3) that has relevance for the later application of immunotherapy. We recently developed criteria (3) by which such a psychiatric syndrome can be classified as having an probable or definitive autoimmune basis. A probable autoimmune origin can be assumed if patients present with a subacute (≤ 3 months) or chronic (≥ 3 months) psychiatric syndrome affecting different symptom clusters such as altered consciousness, disorientation, confusion, memory impairment, obsessive-compulsive behavior, psychosis, catatonia, mood dysfunction, anxiety, behavioral abnormalities (autism, hyperkinetic) and/or sleeping dysfunction. In addition, ≥ 1/9 cardinal features should coincide in these patients, namely the actual or recent diagnosis of a tumor, movement disorder (catatonia, hypo- or hyperkinetic movements), adverse response to antipsychotics or antidepressants, differentially suspected neuroleptic malignant syndrome, severe cognitive dysfunction, altered consciousness, seizures, optic hallucinations, infectious prodrome with fever, aphasia, dysarthria and/or mutism. Furthermore, one of the following criteria must be met: (i) CSF pleoycytosis of > 5 μl white blood cells per μL, oligoclonal bands or intrathecal immunoglobulin G (IgG) synthesis in the CSF, (ii) uni- or bilateral brain abnormalities/ unilateral brain abnormalities on MRI within the temporal lobe or hyperintense lesions outside the limbic system, (iii) EEG abnormalities [spike, spike wave, focal slowing, extreme delta brush, FIRDA or TIRDA (frontal or temporal irregular delta activity)] or (iv) the presence of serum autoantibodies, high Tau-protein or dynamic neurofilament light chains (Nfl) abnormalities related to an acute phase of psychiatric symptoms. A definitive autoimmune origin of psychiatric symptoms exists if patients with a subacute or subchronic probable autoimmune-based psychiatric syndrome present and their CSF reveals IgG class autoantibodies. Immunotherapy is an emerging and important topic in the management of autoantibody-associated syndromes. The administration of immunotherapy is advisable for probable and definitive, but not suspected autoimmune-based psychiatric syndromes, as stated recently in published criteria (3). For this narrative review article on this topic we screened PubMed for the search terms “antibody immunotherapy psychiatry,” “autoantibody immunotherapy psychiatry,” “autoimmune dementia immunotherapy,” “autoimmune encephalitis immunotherapy,” “autoimmune psychosis immunotherapy” or “autoantibody psychosis immunotherapy.” We have included articles in this narrative review that describe case series, observational studies, and randomized placebo-controlled trials between 2010 and 2020 discussing immunotherapy in patients with autoantibodies and psychiatric syndromes in order to attain a reasonably moderate to high evidence level. We therefore decided against focusing on individual case reports published on applying immunotherapy for autoantibody-associated psychiatric syndromes, autoimmune psychosis, and autoimmune dementia. However, for escalating therapy we did look into single case reports and case series, as no other study types have been reported describing such immunotherapies for our patient groups.

## Lessons From Immunotherapy in Autoimmune Encephalitis

Although only one randomized, placebo-controlled trial from Dubey et al. (4) exists for immunotherapy in autoimmune encephalitis, many observational and case series have been published on it. There is consensus among experts concerning first-line therapy encompassing methylpredisolone and/or IVIG (intravenous immunoglobulins) and/or plasmapheresis (PLEX)/immunadsorption (2, 5–7). Second-line immunotherapy consists of rituximab or cyclophosphamide (2, 5–7). If methylprednisolone proves effective in first-line therapy, steroid-saving drugs such as mycophenolate or azathioprine might be used for maintenance therapy (5–7). Alternative therapies are suggested for patients not responding to the aforementioned second-line therapies such as tocilizumab, bortezomib or low-dose interleukin 2 (5–7). For other drugs that depict a novel escalation treatment strategy such as daratumumab, anti-CD38 antibody-depleting plasma cells seem to alleviate clinical symptoms, but more investigations are required for safety reasons (8). Results from a large multicenter observational study in 577 patients with N-methyl-D-aspartate-receptor (NMDAR)-encephalitis recommend an early induction of immunotherapy as it could be highly effective and improve the course of the disease (9). In the study by Titulaer (9), first-line therapy led to clinical improvement in 251/472 (53%) patients and second-line therapy in 125/221 (56%) patients. Randomized placebo-controlled trial in LGI1 (leucine-rich glioma-inactivated 1) – positive encephalitis patients reported a major benefit regarding cognition and seizures after taking IVIGs compared to placebo treatment in 6/8 (75%) patients (4). Another large observational study of 103 patients addressed immunotherapy utilizing corticosteroids alone or in combination with either IVIGs or PLEX, and demonstrated a beneficial effect of early immunotherapy concerning seizure frequency and preventing cognitive impairment (10). In short, the spectrum of therapeutic tools for autoimmune encephalitis is broad and already considered by current national treatment guidelines (11). Here, a step wise escalation of therapy is preferable, but more clinical placebo-controlled randomized trials in autoimmune encephalitis are needed to compare and validate the efficacy and safety of standard first- and second-line therapies. Several immunological agents are now being carefully investigated, such as the proteasome inhibitor bortezomib in a randomized, multicentric, double blind phase II study in patients with severe autoimmune encephalitis (12) as the escalation therapy agent. Additional studies also in patients with antibody-associated psychiatric syndromes must be conducted to investigate the efficacy and safety of such immunotherapeutic approaches.

## Lessons From Autoimmune Psychosis and Immunotherapy

The first trial was conducted in the United Kingdom (UK) and called SINAPP1 (study of immunotherapy in antibody-positive psychosis: feasibility and acceptability (SINAPPS1)] (13) at two sites and in 10 patients. IVIGs were administered in 6/10 patients, whereas 4/10 patients underwent PLEX. Immunotherapy resulted in a marked alleviation of psychotic symptoms assessed by PANSS (Positive and Negative Syndrome Scale) indicating that immunotherapy can be administered rapidly and relatively safely in psychiatric clinics (13). Specific subgroups of patients such as those presenting NMDAR antibodies revealed greater improvement than those with other antibodies such as VGKC complex and GABAAR (13). Following their encouraging results, they have been conducting a randomized, double-blinded, multicentric, placebo-controlled trial (SINAPPS2) since 2017 (to run until 2021) in the UK. One group undergoes immunotherapy consisting of IVIG and rituximab with additional antipsychotic treatment, and the other gets placebo therapy (14). Their primary end point is symptomatic remission for 6 months on PANSS observed for a 12-month period (14). Recent observational studies have indicated that the response to immunotherapy ranges from 67–80% in patients with psychosis (15, 16). The response to immunotherapy has been more successful when the disease duration was short and not previously treatment-refractory (15). Thus, immunotherapy in autoimmune psychosis has been integrated as a therapy recommendation in consensus guidelines according to Pollak et al. (17).

## Lessons From Autoimmune Dementia and Immunotherapy

A large observational study of immunotherapy in 72 patients with presumed autoimmune dementia with progressive and fluctuating cognitive dysfunction based on autoimmunity as revealed by CSF findings and autoantibody detection (18) showed a strong clinical response to immunotherapy including i.v. methylprednisolone, oral prednisolone, i.v. dexamethasone and IVIGs, as well as PLEX in 46 (64%) of all patients (18). In addition, the immunotherapeutic success was dependent on an early treatment onset, but also on the type of antibodies and severity of CNS inflammation. Furthermore, there is evidence that continued immunotherapy is highly relevant, as 77% of patients relapsed after it was terminated (18).

## Autoantibody-Associated Psychiatric Syndromes and Immunotherapy

No placebo-controlled randomized studies exist on the efficacy of immunotherapy in autoantibody-associated psychiatric syndromes in adults. We identified two large cohort observational retrospective case series studies describing immunotherapy in autoantibody-associated psychiatric syndromes, although their autoimmune basis is less certain, and the criteria we recently established for autoimmune-based psychiatric disease were not applied. The rationale for applying immunotherapy in such patients with autoantibody-associated psychiatric syndromes is therefore less clear simply because their psychiatric syndromes have not yet be classified relying on their probable autoimmune origin in light of novel criteria (3). Nevertheless, these two large-scale studies enable an overview of how beneficial immunotherapy is for autoantibody-associated psychiatric syndromes. One such large-scale study by Endres et al. describes immunotherapy in 145 cases of autoantibody-associated psychiatric syndromes (19), showing that immunotherapy was successful in 94% of 126 patients presenting an autoantibody-associated psychiatric syndrome; steroids proved useful in 106 of 119 (89%) of patients, and 55/126 patients (44%) underwent more than one immunomodulatory treatment, yielding an 89% improvement rate. IVIGs were applied in 55 of 126 (43%) of patients, whereas 42 of 126 (33%) received plasma exchange therapy (19). Immunotherapy was more beneficial in those patients with autoantibody-associated psychiatric syndromes who presented with autoantibodies against membrane-surface antigens [77 of 80 patients (88%)] compared to those with autoantibodies against intracellular antigens [6/15 patients (40%)] (19) resembling the findings on patients with autoimmune encephalitis (20–22). Another large cohort study from the same working group (23) investigated the occurrence of autoantibody-associated psychiatric disease in patients with schizophrenic and affective psychosis. Thirteen of 24 antibody-positive patients in this large cohort received immunotherapy, and 87% exhibited a good response to immunotherapy which had azathioprine, methotrexate, mycofenolat mofetil, and rituximab. Taken together, these large observational studies (19, 23) deliver a low, but consistent evidence level that in those patients with psychiatric syndromes and proven autoantibodies, immunotherapy succeeded in 87–94% of patients (*n* = 139). The evidence from these large retrospective case studies supports the usefulness of immunotherapy (Table 1). A recent study of McGinty (31) seems to be of major interest regarding the application of immunotherapy, as his study revealed a good outcome in autoimmune-epilepsy patients presenting autoantibodies, but no other indices of an autoimmune encephalitis. However, although his work did not address autoantibody-associated psychiatric syndromes, it does question the requirement of an immunotherapy for clinical syndromes associated with autoantibodies in patients not presenting any proof of an underlying encephalitis. Thus, more investigations are necessary comparing the outcomes of patients with autoantibody-associated psychiatric syndromes but no proven encephalitis, with and without immunotherapy.

**Table 1:** Immunotherapy in autoimmune encephalitis and autoimmune-based psychiatric syndromes.

**Drug**	**Mechanism**	**Evidence for autoantibody associated psychiatric syndrome**	**Evidence for autoimmune encephalitis**	**Dosage**	**Pro**	**Contra**
**First line therapy**						
Steroids	Impair the travel of immune cells to the CNS Depletion of CD4+T-cells Inhibition of macrophages Inhibiting proinflammatory cytokines Induction of apoptosis of immune cells Inhibiting inflammation	(15–19, 23)	(2, 4–7, 9, 10, 21, 22)	1 g for 3 d or 5 d i.v. m over 6 m	Cheap easy to apply	Induction of depression, mania or sleep dyfunction Infections Blood cell changes
IVIG	Immunodulation of cytokines, B-cells, macrophages, T-cells, cell adhesion proteins, complement system Anti-inflammatory effects	(13, 16–19, 23)	(2, 4–7, 9, 10, 21, 22)	0,4 g/kg/d for 5d, after 1/ 5w	Expensive Allergy If steroids do not work or side effects or contraind. Against steroids	IgA deficiency Hypersensitivity Renal failure Headache Nausea
PLEX	Elimination of serum abs	(13, 17–19, 23)	(2, 5–7, 21, 22, 24)	1 d+, 1 d-for 5–7 cycles	Depletion of serum abs	Syndrome with edema Severe liver disease Coagulopathy Hypotension Pneumothorax
**Second line therapy**						
Rituximab	Depletes CD20 B-cells	(13, 16, 19, 23)	(2, 5–7, 10, 22, 25)	375 mg/m^2^ w for 4 w	Failure of first line therapy -IgG4 subclass present	Severe infections Hemorrhage Myalgia Peripheral edema PML
Cyclophosphamid	Covalent attachment on alkylgroups to DNA Inhibition of protein synthesis Suppression of Tregs	(19, 23)	(2, 5–7, 22)	750 mg/m^2^ 1 x m for 3–6 m	T-cell mediated disease	Myelosuppression Hemorrhagic cystitis Alopecia Nausea Liver disease
**Maintenance therapy**						
Azathioprin	Inhibits purin metabolism Reduction in DNA, RNA for blood cell synthesis	(16, 23)	(2, 5–7)	1.5 mg/kg 1 x d td: 2–3 mg/kg/d	Saving steroids	Diarrhea Alopecia Lymphoma Fever Liver disease
Mycofenolat mofetil	Reverse, selective inhibition of IMPDH, enzyme of purinbiosythesis Thereby depletion of T-and B-cell dependent guanosine nucleotides	(23)	(2, 5–7)	500 mg 2 x d, td: 1000 mg	Saving steroids	Hypertension Infections Myelosuppression PML Allergy
**Escalation therapy**						
Tocilizumab	Monoclonal antibody Inhibition of IL-6 binding to its receptor (IL-6R) blockage of IL−6 based inflammation on T – and B-cells, for example, impair Tregs	—	(25, 26)	4 mg/kg, escalating to 8 mg/kg/m	T-cell mediated disease	Severe infections Hypertension Gastritis Hypercholesterinemia Transaminasis elevation
Interleukin 2	Binding to IL−2 R expression by lymphocytes (activated CD4+ and CD8+ T -cells) Promotion of T-cell growth factor Improves balance between Tregs and effector T-cells	—	(27)	1.5 million IU/ d, 4x s.c. every 3 w		Flush diarrhea Liver dysfunction Tachykardia Fever
Bortezomib	Proteasom inhibitor Interference with the NF -κB and ubiquitin proteasome pathway	—	(28–30)	1.3 mg/m^2^ i.v. d 1, 4, 8, 11	B-cell mediated disease	Anorexia Dehydration Hypotonia Neuropathy Myalgia
Basiliximab	Affects plasma B-cells -myalgia with consecutive apoptosis Glycoprotein Reduce cytotoxic T-cells Blockage of alpha chain IL-2 R	—	40	2x20 mg 1 d 1x 20 mg 4 d	T-cell mediated disease	ypertension Constipation Peripheral edema Urinary tract infections Nausea

*abs, antibodies; CD, chain of differentiation; CNS, central nervous system; contraind, contraindications; d, days; DNA, deoxyribonucleic acid; g, gram; IgG4, immunoglobulin G4 subclass; IL-2, interleukin 2; IL-2 R, interleukin 2 receptor; IL-6, interleukin 6; IL-6 R, interleukin 6 receptor; IMPDH, Inosine-5'-monophosphate dehydrogenase; i.v., intravenous; IVIG, intravenous immunoglobulins; kg, kilogram; m, month; mg, milligram; PLEX, plasmapheresis; PML, progressive multifocal leukencephalopathy; RNA, ribonucleic acid; s.c., subcutaneous; td, targeted dosage; Tregs, regulatory T-cells; w, week. Cave: this table depicts the subjective view of the authors with pros and cons, mechanisms or described study evidence that does not claim to show the complete possibilities of mechanisms, evidence, pros or cons*.

### First-Line Immunotherapy in Autoimmune-Based Psychiatric Syndromes

**Figure 1:**
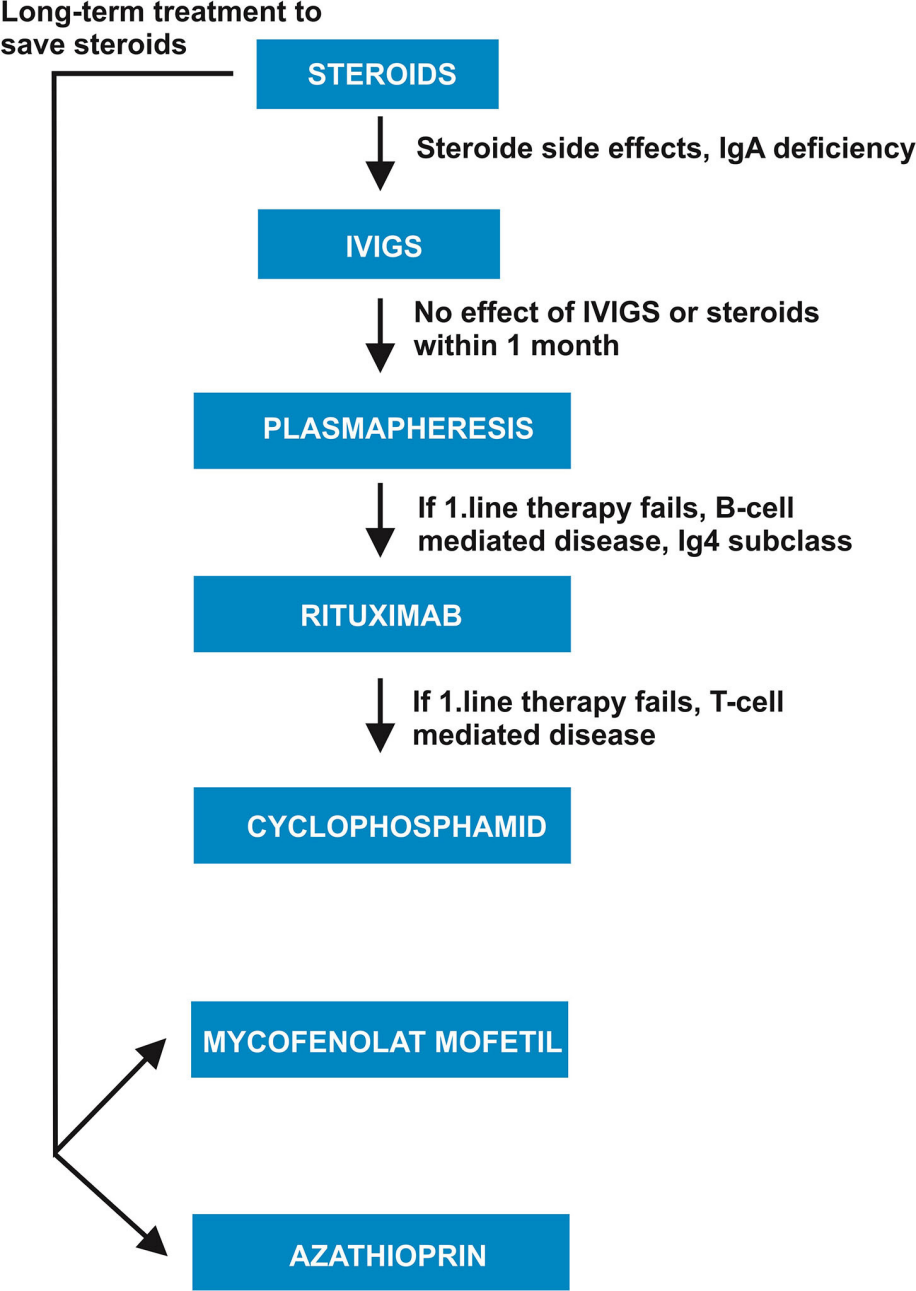
Clinical pathway for selecting immunotherapy for autoimmune-based psychiatric syndromes. IVIGS/s, intravenous immunoglobulins; IgA, immunoglobulin A; IgG4 subclass, immunoglobulin G4 subclass.

In line with the aforementioned studies (19, 23), we recommend immunotherapy (Figure 1 and Table 1) for patients presenting probable or definitive autoimmune-based psychiatric syndromes according to this therapeutic pathway (Figure 1 and Table 1): first-line immunotherapy should include steroids (i.v. or oral), IVIGs, immunoadsorption and/or PLEX. The evidence level for IVIGs in autoimmune encephalitis relies on one randomized trial (4), but the other therapeutic options rely on an evidence level originating from case series and large observational studies (9, 10). We recommend immunotherapy following the careful assessment of specific comorbidities, potential side effects, and patients' contraindications. Corticosteroids are often the first choice, whereas IVIGs and PLEX are the second option, especially if steroids have been ruled out due to their side effects. Steroids exert multiple effects on the immune system and might thus act on many sites concurrently, such as cytokine modulation (32, 33), depleting immune cells such as CD4+ T-cells (34), and inhibiting immune-cell activity such as macrophagic function (35). For the above-mentioned immunologic agents' potential disease mechanisms, see Table 1. Considering corticosteroids' mild-to-moderate effects on autoantibody-producing B-cells and blood autoantibodies, combining them with other agents depleting those cells and blood autoantibodies such as PLEX seems advantageous for patients with severe symptoms, but it demonstrates no response to steroids or antibodies against membrane antigens. Another issue to consider when applying steroids: certain populations of psychiatric patients may be more sensitive to additional aggravated or induced depressive, manic, sleep dysfunction, or psychotic symptoms. Therefore, careful monitoring is mandatory when psychiatric patients are undergoing immunotherapy. In addition to steroids, IVIGs are a first-line option to choose for patients presenting the aforementioned side effects from steroids or their contraindications (Figure 1 and Table 1). IVIGs are plasma collected from diverse patients, containing various antibodies suitable for hindering pathogenic T-cells (while they travel through the central nervous system) (36), and for producing anti-inflammatory effects (37). To prevent any anaphylactic reaction, only immunoglobulin A (IgA)-deficient patients should not be given IVIGs (Figure 1 and Table 1). PLEX is invasive, and thus the potentially last therapeutic option for first-line immunotherapy. It works by removing blood autoantibodies. First-line immunotherapy with IVIGs and or methlyprednisolone should have failed before applying PLEX. Immunoadsorption is equivalent to PLEX, and seems to have reduced additional CSF antibodies in single case series in patients with autoimmune encephalitis (24).

### Second-Line Immunotherapy in Autoimmune-Based Psychiatric Syndromes

Second-line therapy should encompass rituximab or cyclophosphamide and is required if first-line therapy fails to show a response within 1 month in autoimmune-based psychiatric syndromes (Figure 1 and Table 1). The evidence level for autoimmune-based psychiatric syndromes is much lower when relying on single cases, that is, those in the retrospective cohort studies of Endres et al. (19, 23). The evidence for immunotherapy for patients with autoimmune encephalitis also relies on observational studies and case series. Rituximab is especially beneficial for autoimmune encephalitis patients, as shown in a study by Thompson et al. (10). As second-line immunotherapy, rituximab should be chosen if autoantibodies are playing a probable immunopathological role (as suspected in NMDAR-positive psychiatric symptoms), as rituximab mainly depletes B-cells via possible mechanisms like (1) cellular toxicity, (2) humoral response activation such as the complement cascade, and/or (3) apoptosis (38–40). It thus makes sense to apply rituximab in those patients with autoantibody-based psychiatric syndromes and proven membrane-surface autoantibodies such as the NMDAR autoantibody. Rituximab is further recommended if the main immunoglobulin (IgG) subclass detected consists of IgG4 (as proven, for example, in CASPR2-positive or IgLON5-positive encephalitis (41, 42). Cyclophosphamide should be the second option after rituximab due to its serious side effects, that is, hemorrhagic cystitis or myelosuppression. However, as it specifically suppresses lymphocyte proliferation, it is also suitable for use in probably T-cell-mediated diseases, eg, autoimmune-based psychiatric symptoms associated with antibodies against intracellular antigens.

### Maintenance Immunotherapy in Autoantibody-Based Psychiatric Syndromes

In individual cases of autoantibody-associated psychiatric syndromes, mycophenolate mofetil and azathioprine proved to improve outcome (19). These agents are suggested for maintenance therapy in steroid-responsive patients. We also recommend mycophenolate mofetil or azathioprine to reserve steroid application for long-term treatment (Figure 1 and Table 1).

### Escalation Immunotherapy in Autoantibody-Based Psychiatric Syndromes

Alternative or escalation therapy could entail poorly-investigated substances that have proven beneficial in individual case series or observational studies with specific antibodies such as bortezomib in NMDAR-positive encephalitis (28), tocilizumab as an interleukin-6 antibody in patients with NMDAR, LGI1 (leucine-rich glioma-inactivated 1) and amphiphysin antibodies (26) and low-dose interleukin 2 (27). Note, however, that the evidence level for this kind of immunotherapy is low; furthermore, there is currently no evidence for its administration for autoantibody-associated psychiatric syndromes. Basiliximab is a potentially beneficial interleukin 2 receptor antibody in GAD65-associated encephalitis, as a reduction in cytotoxic CD8+ T-cells in the CSF has been demonstrated (43). Thus, its application makes sense as experimental therapy for autoimmune-based psychiatric syndroms associated with GAD65 or other antibodies against intracellular antigens. Some agents deserve mention in detail as alternative therapies, although the clinical evidence of benefit is low due to only small case series in autoimmune-encephalitis patients. Tocilizumab is one of these promising candidates, as it impairs interleukin 6-dependent inflammation and inhibits the development of regulatory T cells that play a major role in autoimmune-based neuronal damage (44–46). It thust seems particularly important to test this substance, especially in those patients with autoimmune-based psychiatric syndromes and proven autoantibodies directed against intracellular antigens. Low dose interleukin 2 is another intriguing candidate, as interleukin 2 regulates the activity of T-cells and provides tolerance over autoimmunity (47) in autoimmune encephalitis. The proteasome inhibitor bortezomib is another agent to administer in patients presenting severe NMDAR-associated psychiatric symptoms due to autoimmune encephalitis, as several studies have demonstrated (28–30). We suspect that bortezomib might prove beneficial in autoimmune-based psychiatric syndromes with autoantibodies against membrane-surface antigens. Another intriguing candidate is the humanized anti-CD19 antibody currently being investigated in the NCT04372615 trial in NMDAR encephalitis patients (phase II) as a potential escalating therapy for autoimmune encephalitis in conjunction with suspected B-cell-mediated disease. A recent study showed that an antibody-negative limbic encephalitis patient often revealed a strong CD19+ B-cell response in serum and CSF through fluorescence cell-sorting analysis (25). In conclusion, as there is currently no evidence for applying these types of experimental escalation therapy for autoimmune-based psychiatric syndromes, more investigations must be carried out to verify their safety, tolerability, and efficacy in our targeted patient collective.

## Limitations

The present review's main limitation is that the sources of evidence on the administration of immunotherapy for autoantibody-associated psychiatric syndromes is low, and relies on a handful of observatory studies (15, 16, 19, 23) and guidelines (17). The experimental therapies suggested for escalation therapy are derived only from single case reports or case series (26–28, 30, 43, 48) for patients with autoimmune encephalitis. We wish to highlight the low-to-moderate evidence levels, as randomized, placebo-controlled trials are still lacking, as are meta-analyses of patients with autoantibody-associated psychiatric syndromes in general, and those with specifically autoimmune-based psychiatric syndromes.

## Ethical Aspects

Immunotherapy in patients with autoimmune-based psychiatric syndromes is an optional and experimental therapy relying on an evidence level IV following case control and cohort studies. Thus, the patient should be well-informed, provide written consent and agree to the treatment on an individual therapeutic trial basis.

## Conclusions

Taken together, autoimmune-based psychiatric syndromes have emerged as a novel category of disorders. Immunotherapy is a therapeutic option in autoimmune encephalitis, autoimmune psychosis and autoimmune dementia that should be initiated also in autoimmune-based psychiatric syndromes in analogy to autoimmune encephalitis and autoantibody-associated psychiatric syndromes early to improve the clinical outcome. The early onset of immunotherapy is likely to be relevant to prevent permanently cognitive and behavioral dysfunctions. Lessons from autoimmune encephalitis and psychosis have revealed specific immunotherapeutic agents termed first- and second-line immunotherapies also applied in patients presenting autoantibody-associated psychiatric syndromes. Furthermore, to stabilize and support these patients over the long term, other immunotherapies referred to maintenance or alternative therapy might be administered to severely-affected or drug-refractory patients. Further randomized, placebo-controlled trials are needed to evaluate immunotherapies in patients with autoimmune-based psychiatric syndromes and to prove their efficacy, inferiority or superiority over other immunotherapies. To accelerate and improve the diagnosis-based treatment of autoantibody-associated psychiatric syndromes, an individual, fundamental diagnostic approach must be taken involving CSF, EEG and MRI investigations at psychiatric in- and outpatient institutions and hospitals.

## Author Contributions

NH conceived the review and wrote the review draft. CT critically revised the manuscript for important intellectual content. All authors have read and agreed to the published version of the manuscript.

## Conflict of Interest

The authors declare that the research was conducted in the absence of any commercial or financial relationships that could be construed as a potential conflict of interest.
